# Distinguishing migration from isolation using genes with intragenic recombination: detecting introgression in the *Drosophila simulans* species complex

**DOI:** 10.1186/1471-2148-14-89

**Published:** 2014-04-24

**Authors:** Miguel Navascués, Delphine Legrand, Cécile Campagne, Marie-Louise Cariou, Frantz Depaulis

**Affiliations:** 1 UMR 7625 Écologie et Évolution (CNRS/École Normale Supérieure/Université Pierre et Marie Curie), Paris, France; 2 INRA, UMR1062 CBGP, F-34988 Montferrier-sur-Lez, France; 3 USR 2936, Station d’Écologie Expérimentale du CNRS, 09200 Moulis, France; 4 UPR 9034 Évolution, Génomes et Spéciation, CNRS, avenue de la Terrasse, 91198 Gif sur Yvette, France; 5 Laboratoire d’Écologie, École Normale Supérieure, 46 rue d’Ulm, 75230 Paris cedex 05, France

**Keywords:** Shared polymorphism, Recombination, Hybridization, Gene flow, Incomplete lineage sorting, *Drosophila simulans* complex

## Abstract

**Background:**

Determining the presence or absence of gene flow between populations is the target of some statistical methods in population genetics. Until recently, these methods either avoided the use of recombining genes, or treated recombination as a nuisance parameter. However, genes with recombination contribute additional information for the detection of gene flow (i.e. through linkage disequilibrium).

**Methods:**

We present three summary statistics based on the spatial arrangement of fixed differences, and shared and exclusive polymorphisms that are sensitive to the presence and direction of gene flow. Power and false positive rate for tests based on these statistics are studied by simulation.

**Results:**

The application of these tests to populations from the Drosophila simulans species complex yielded results consistent with migration between D. simulans and its two endemic sister species D. mauritiana and D. sechellia, and between populations D. mauritiana on the islands of the Mauritius and Rodrigues.

**Conclusions:**

We demonstrate the sensitivity of the developed statistics to the presence and direction of gene flow, and characterize their power as a function of differentiation level and recombination rate. The properties of these statistics make them especially suitable for analyzing high-throughput sequencing data or for their integration within the approximate Bayesian computation framework.

## Background

Assessing gene flow is essential for any study of speciation or local adaptation, as gene flow is a force counteracting those processes. Classic models of population genetics consider the source of genetic differentiation between populations (*F*_*ST*_) to result either from an equilibrium between migration and drift (island model) or from drift since the time of divergence (isolation model). Thus, population geneticists would estimate migration rates [1] or time of divergence [2] from *F*_*ST*_ based on their (expert) opinion or other non-genetic evidence that their study system fitted best to one of the two alternative models. The first attempt to distinguish between both scenarios using genetic data was made by Wakeley [3], who noted that the variance of pairwise differences was expected to be higher under the migration model and proposed a test statistic based on this prediction. However, a test based on the variance of pairwise differences shows low power [3], is highly influenced by recombination, and has seen only limited implementation. A significant advance was the development of likelihood-based methods under the isolation-with-migration model e.g. [4]. In this approach, the model consists of two populations that diverge from an ancestral population and exchange migrants, and the values of these parameters (migration rate and divergence time) are inferred. This is currently the most widely used method to study genetic differentiation between a pair of populations or species (see [5] for a review) and is implemented in IMa [6]. The model implemented in IMa assumes the absence of intragenic recombination and the violation of this assumption can produce substantial bias in the estimates from this analysis [7]. A framework to specifically analyse recombining genes is essential as nuclear genes, which are widely used in studies related to divergence and speciation, are subject to recombination. In order to overcome this limitation, likelihood-free methods have also been considered e.g. [8], in which data are summarized by a set of statistics, and the likelihood is approximated by a distance metric between the observed summary statistics and summary statistics simulated from the model. Thus, loci with intragenic recombination can be simulated under the isolation-with-migration model to approximate the likelihood of the parameter values. Similarly, other recent approaches based on summary statistic or the site frequency spectrum e.g. [9,10], use coalescent simulations with recombination to account for linkage among markers. Recombination generates different linkage disequilibrium patterns depending upon the presence or absence of gene flow ([11]; see next section for details); so genes with recombination potentially provide additional information about genetic exchange. However, because these approximate-likelihood methods reduce the data to a set of summary statistics, information on linkage disequilibrium between polymorphic sites is usually lost (for instance, summary statistics used in MIMAR [8] do not contain this type of information), and any additional information provided by intragenic recombination cannot be exploited. Among the latest related statistical developments, it is worth noting the PAC-likelihood (Product of Approximate Conditional probabilities) method [12] and a method based on shared haplotype lengths [13], both of which explicitly exploit the spatial arrangement of polymorphism within sequences to make inferences under the isolation-with-migration model.

In this work we propose summary statistics that contain information about the presence and direction of gene flow as a result of intragenic recombination, and we describe their properties in the form of statistical tests for the detection of gene flow (note, however, that their use is not necessarily limited to such tests). In order to provide an empirical example, these statistics were calculated in a sample of eleven loci sequenced from populations of the *Drosophila simulans* species complex (*D. simulans*, *D. sechellia* and *D. mauritiana*, Additional file 1: Figure S4), which is one of the most documented models used for speciation and evolution [14-16]. The generalist *D. simulans,* which probably evolved on Madagascar [17], has recently extended its distribution globally, is now a semi-domestic species, exhibiting strong genetic differentiation between ancestral and derived populations [18-20]. In contrast, the endemic *D. sechellia*, confined to the Seychelles archipelago, presents all the characteristics of a island-syndrome species, being strictly specialized on the ripe fruit of the otherwise toxic *Morinda citrifolia*[21], with low reproductive output [22] and presenting limited genetic diversity [23-25]. Despite its fragmented distribution, *D. sechellia* does not exhibit strong population structure [25], but rather a local pattern of genetic exchange between neighboring islands [26]. These features strongly contrast with those of its sister species, the island endemic *D. mauritiana*, which is geographically and genetically highly structured into two populations: the expanding population of Mauritius Island and the population of Rodrigues Island, 600 km to the east of Mauritius, which is smaller and at equilibrium [27]. Interestingly, *D. simulans*, *D. sechellia* and *D. mauritiana* are incompletely reproductively isolated, and can produce fertile female F1 hybrids (males are sterile) [28,29]. However, the question of interspecific hybridization in nature, and its frequency, is unresolved. The existence of shared polymorphisms between these species may thus result from introgression due to secondary contact, but could also be due to ancestral polymorphism shared among these recently diverged species.

## Results and discussion

### Spatial arrangement of polymorphism with recombination and gene flow

**Figure 1:**
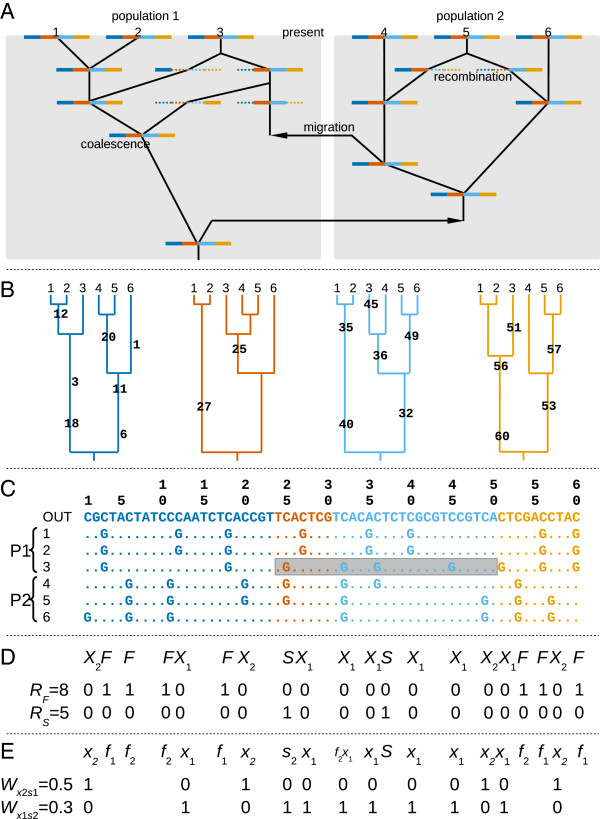
**Ancestral recombination graph, genealogies and polymorphisms for samples taken from two populations exchanging migrants.** Colors represent the different sections defined by recombination events. **A.** Ancestral recombination graph (lines in black) of six gene copies (1–6) sampled from two populations (represented by background blue shade). Present is represented at the top and past at the bottom (the time is not scaled). Each gene copy is represented by a four-colour bar; within which, dotted sections represent the length of the gene that will not leave descendants in the sample. **B.** Genealogies of the six gene copies (1–6) for each of the segments (represented by different colours) delimited by the recombination events. Mutation events are represented on the branches together with their position in the alignment. **C.** DNA sequence alignment of the six gene copies (1–6) plus an outgroup (OUT) sequence used to inferr the ancestral state of mutations. An introgression block (or migrant tract) is marked with a grey rectangle. **D.** Categories of polymorphic sites [30] found in the alignment (fixed differences, *F*, exclusive polymorphisms of each population, *X*_1_ and *X*_2_, and shared polymorphisms, *S*). The randomness of the order of categories is assessed with two statistics based on the number of runs (*R*, see main text for more details). **E.** Categories of polymorphic sites taking into account the direction of mutation [in practice, by means of an outgroup [38] and whether the derived state is fixed in population 1 (*f*_1_, *f*_1_*x*_2_) or in population 2 (*f*_2_, *f*_2_*x*_1_). The spatial clustering of polymorphic site categories along the alignment is assessed with the *W* statistic (see main text for details).

The segregating sites from a sample of sequences taken from two populations can be divided into four categories [30] (see Figure 1): shared polymorphic sites (*S*), which are polymorphic in both populations; fixed polymorphic sites (*F*), which are fixed differences between the two populations (i.e. monomorphic for different alleles within both populations); exclusive polymorphic sites of population P1 (*X*_1_), which are sites polymorphic in population P1 and monomorphic in population P2; and exclusive polymorphic sites of population P2 (*X*_2_).

In order to illustrate the problem at hand, we will consider two extreme models: the isolation (two populations diverging from an ancestral one) and the migration (two populations only connected by gene flow) models. In the case of the isolation model, the origin of *S* sites are polymorphisms from the ancestral population that have survived drift in both populations since the time of divergence, a process often referred to as incomplete lineage sorting e.g. [31]. In the case of the migration model, shared polymorphism is caused by (possibly recent) exchange of alleles between the populations. It has been argued [11] that *S* sites had more time to recombine with other sites under the isolation model than under the migration model, and, therefore, stronger linkage disequilibrium (LD) between *S* sites within each population was expected with the presence of gene flow. However, a preliminary study found that classical measures of LD have little power for the detection of gene flow. While recent gene exchange leaves little time for recombination to act it also leaves little time for (migrant) allele frequencies to increase. Since LD measures are very sensitive to allele frequencies, detecting a significant association between rare migrant alleles is challenging (F. Depaulis, unpublished results). In summary, there are two balanced effects: introgressed alleles may lead to greater LD because they are recent, leaving little time for recombination to occur, however they also tend to be rare, which does not favour high LD values. Note, however, that LD measures might be used to detect admixture [32], i.e. a population originating from the mixture of individuals from different populations, because the targeted pattern is not expected to be overly influenced by low allele frequencies.

Here we argue that gene flow creates some patterns of LD distinct from those created under the isolation model (for an equivalent differentiation level) and which require non-standard measures of LD to reveal them. In the migration model, the fate of a migrant chromosome is to be fragmented by recombination into “introgression blocks”, i.e. segments of DNA of migrant ancestry and untouched by recombination since the migration event (named “migrant tracts” in ref. [33]). Some introgression blocks may be lost by drift, while others may persist. Figure 1 represents a simplified genealogy with migration and recombination (i.e. an ancestral recombination graph; Figure 1A) and a resulting introgression block (Figure 1C, within a grey rectangle). A set of aligned sequences can be divided into segments delimited by the recombination breakpoints (i.e. there would be as many segments as recombination events plus one, represented in Figure 1 with different colours). A segment of the alignment containing an introgression block may contain shared polymorphisms but will rarely contain any fixed difference (except in less likely scenarios, such as when the last lineage with an ancestral state in one population sample migrates to the other population, leaving the source population sample fixed for the mutant allele and the recipient population sample fixed for the ancestral allele). Conversely, a segment of the alignment that does not contain any introgression blocks may contain *F* sites (lineage sorting is complete) but cannot contain *S* and *F* sites together (Figure 1D). Therefore, *F* and *S* sites along the alignment are expected to be segregated into a small number of groups.

An alignment can be summarized as a sequence that represents the order of the different categories of polymorphic sites (Figure 1D; ignoring *X* sites results in a sequence of two elements “*FFFFSSFFF*” or, in binary coding, “000011000”). We expected the order of elements in this sequence to depart more drastically from randomness in models with migration. The runs test [34] is a statistical test for the randomness in the order of elements of two categories along a sequence. A run is defined as a maximal segment of consecutive elements of the same type (e.g. the sequence “000011000” contains three runs). A low number of runs (*R*) within a sequence indicates that identical elements appear in clusters along the sequence (e.g. three runs on sequence “000011000” vs. seven runs on sequence “010010010”). Ideally, we would like to apply the test to the sequence of *F* and *S* sites, but it is unlikely that both types of sites are present in the same DNA alignment for a large set of parameter values combinations (differentiation level and recombination rate). Therefore, we considered the sequence with the four categories of sites (*F*, *S*, *X*_1_ and *X*_2_) and pooled categories of sites, reducing them in two categories. Two combinations were considered: *F* sites vs. *S*, *X*_1_ and *X*_2_ (e.g. Figure 1D; statistic *R*_*F*_) and *S* sites vs. *F*, *X*_1_ and *X*_2_ (e.g. Figure 1D; statistic *R*_*S*_). Since *F* and *S* sites are not often found together, this approach allows testing for either the clustering of *F* sites (*F* vs. *X*) or *S* sites (*S* vs. *X*), whichever are found in the alignment. We predict that values of run statistics (i.e. number of runs, *R*_*F*_ and *R*_*S*_) will be lower under models with migration than under models of pure divergence.

**Figure 2:**
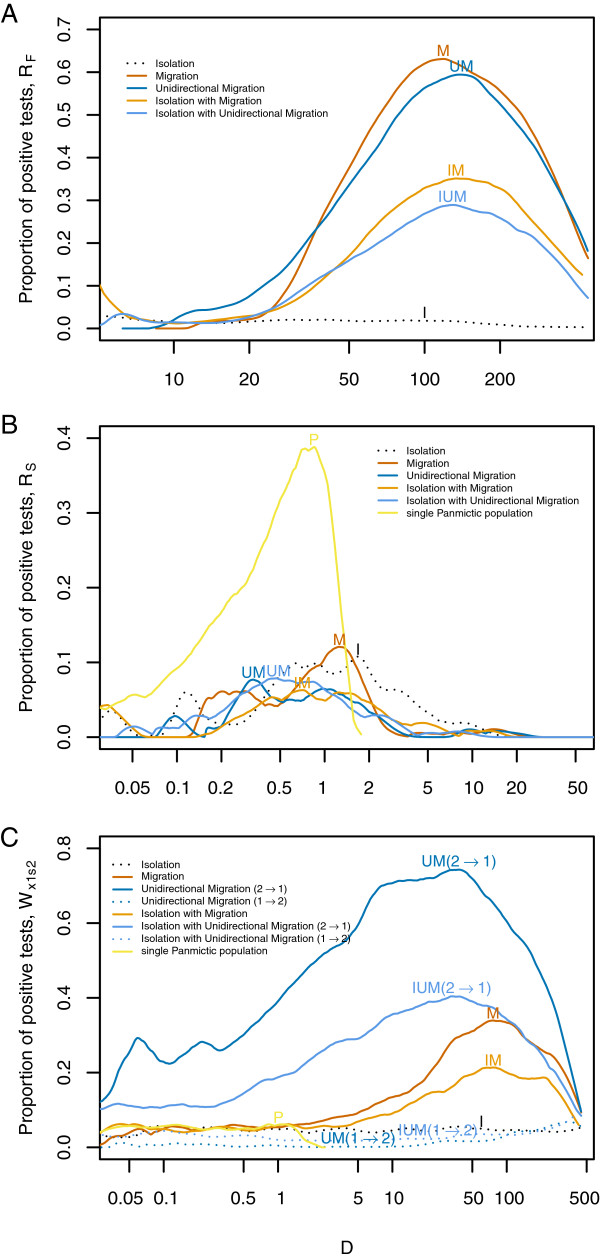
**Proportion of significant tests in simulated data.** 14,000 coalescent simulations, over the whole range of genetic differentiation, were performed for each model: isolation (I, black), migration (M, red), unidirectional migration (UM, dark blue), isolation with migration (IM, orange), isolation with unidirectional migration (IUM, pale blue) and a single panmictic population (P, yellow; note that this line is limited to low values of *D* and thus is missing in panel **A)**. Continuous lines indicate the proportion of significant tests for the models with presence of migration (for *R* statistics) or presence of unidirectional migration from P2 to P1 (for *W* statistics), i.e. they indicate the power of the test (dark and pale blue orange continuous lines have unidirectional migration from P2 to P1). Dotted lines indicate the proportion of significant tests under the models without migration (for *R* statistics) or without migration from P2 to P1 (for *W* statistics), i.e. they indicate the false positive rate (dark and pale blue dotted lines have unidirectional migration from P1 to P2). Proportion of significant tests is estimated as a function of the level of differentiation measured by *D*[35]*.***A.***R*_*F*_ statistic, **B.***R*_*S*_ statistic, **C.***W*_*x1s2*_ Statistic.

**Figure 3:**
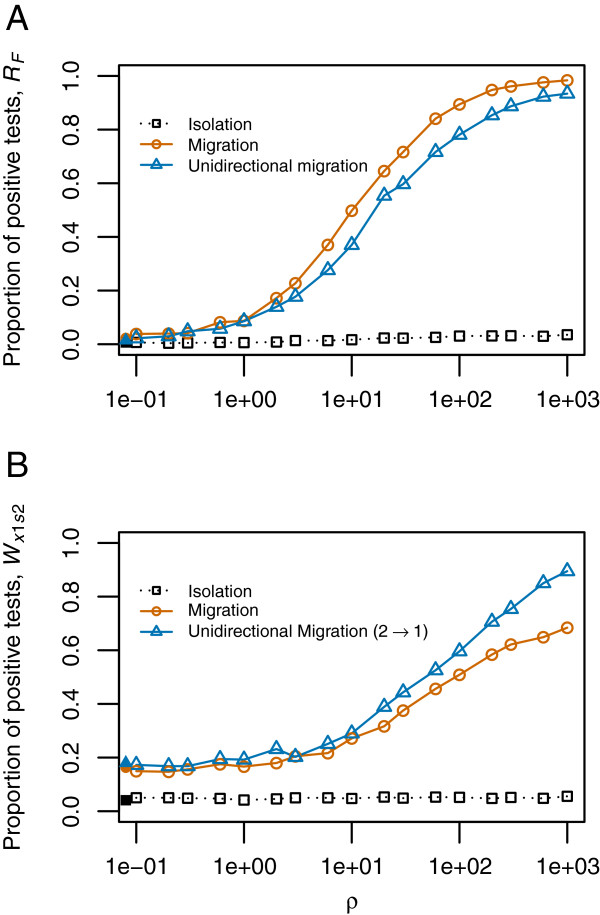
**Effect of recombination rate on the proportion of significant tests.** 1,000 coalescent simulations were performed for each value of recombination parameter ρ an each model: isolation (black), migration (red) and unidirectional migration (blue). Continuous lines indicate the proportion of significant tests under the models with the presence of migration (for *R* statistics) or presence of unidirectional migration from P2 to P1 (for *W* statistics), i.e. they indicate the power of the test. Dotted lines indicate the proportion of significant tests under the models without migration (for *R* statistics) or without migration from P2 to P1 (for *W* statistics), i.e. they indicate the false positive rate. Proportion of significant tests is estimated as a function of recombination rate*.* Filled symbols report the values for ρ = 0 and are represented at an arbitrary position of the x-axis. **A.***R*_*F*_ statistic, **B.***W*_*x1s2*_ Statistic.

Pseudo-data generated by coalescent simulations confirmed our prediction for the *R*_*F*_ statistic. Scenarios with migration resulted in low *R*_*F*_ values, indicating segregation between fixed differences and exclusive (and shared, whenever present) polymorphisms along the alignment. However, the behaviour of the *R*_*F*_ statistic is highly dependent on the differentiation between the two populations, requiring high differentiation (*D* > 20 in our simulations; where *D* stands for Nei’s net distance [35], see Methods) to observe a difference between models with and without migration (Figure 2A). From *D* > 100, power starts to decrease, which is expected as highly differentiated populations are connected by migration events that are distantly spaced in time, so recombination has time to break up associations between alleles. The proportion of false positives (significant *R*_*F*_ values) under the isolation model remains around the nominal value (0.05) for any *D* value (Figure 2A). The distinct behaviour of the *R*_*F*_ statistic under the isolation model and the models with migration indicates that it can be used to tackle the problem in hand under a large range of conditions. Nevertheless, a minimum recombination rate (*ρ* > 1, i.e. some recombination events are necessary) is required for the segregation between *F* and *X* sites, and the signal for this segregation becomes stronger with increasing recombination rate (Figure 3A). In theory, it should start to decrease with recombination at some point (in the extreme, completely independent sites should have no excess of LD), but this was not observed in the range of recombination rates that could be assessed in practice. In contrast with the results for *R*_*F*_, the detection of migration using the clustering of shared polymorphisms (*R*_*S*_) was unsuccessful (Figure 2B). Examining the actual *R*_*S*_ values (Additional file 1: Figure S5B) reveals that highly differentiated populations (*D* > 50) connected with migration do present clusters of shared polymorphisms (low *R*_*S*_ values), but divergent populations do not maintain ancestral polymorphisms at such levels of differentiation. However, shared polymorphisms in populations with lower levels of divergence have a stronger clustering of *S* sites than models with migration. Intuitively, we can expect that low divergence times still retain some clustering of *S* sites in the Isolation model. This is because there has not been enough time for recombination to disrupt allele association, while the presence of high gene flow causes a constant introduction of migrant haplotypes affecting the whole region of the alignment (i.e. there is overlap of introgression blocks along the whole alignment, thus no segregation of *S* sites). Given the different behaviour (though unexpected) of the *R*_*S*_ statistic for the different models, it may be still useful to distinguish the models in another inferential context (e.g. as a summary statistic in an approximate Bayesian computation analysis).

### Spatial arrangement of polymorphism with recombination and unidirectional gene flow

Additional categories of segregating sites can be defined if we consider the distribution of the ancestral and derived alleles between the populations [36]. In practice, the ancestral or derived status of alleles is inferred by the use of an outgroup (Figure 1E). Fixed differences (*F*) can then be further separated into *f*_1_ sites, where the derived allele is fixed in P1, and *f*_2_ sites, where the derived allele is fixed in P2. Among the exclusive polymorphisms of P1 (*X*_1_), we can define the category *f*_2_*x*_1_ for cases where the derived state is fixed in P2 (the remaining *X*_1_ sites will be denoted *x*_1_ to maintain the nomenclature in [36]; similarly, *f*_1_*x*_2_ and *x*_2_ sites may be defined. While in [36] all shared polymorphism were considered in the same category, we will distinguish between *s*_1_ sites for shared polymorphisms with a higher frequency of the derived allele in P1 than in P2, and *s*_2_ for shared polymorphism with higher frequency of the derived allele in P2.

Consider a stretch of the alignment, delimited by recombination events, unaffected by migration (without any introgression block in any individual) that contains only four types of variable site category: *f*_1_, *f*_2_, *x*_1_ and *x*_2_ (assuming that both populations share a common origin but have been separated long enough for full lineage sorting and for new mutations to have occurred within each population). After a migration event from P2 into P1, an introgression block is introduced into the stretch of alignment considered. Thus, all *f*_2_ sites become *f*_2_*x*_1_ and all *f*_1_ sites become *x*_1_. Some *x*_2_ sites may remain *x*_2_ (migration of the ancestral allele; also, more rarely, some *x*_2_ may become *f*_2_ as discussed above) while others may become *S* sites (migration of the derived allele). Those shared polymorphisms will often belong to category *s*_2_, because the mutation is older than the migration event and thus, had more time to increase in frequency in P2 than in P1. Therefore, migration from P2 to P1 will tend to produce clusters of *x*_1_, *f*_2_*x*_1_ and *s*_2_ (Figure 1E), while *x*_2_ sites will be present along the entire alignment, both within the stretch containing introgression blocks, and outside them. Conversely, migration from P1 to P2 will produce clusters of *x*_2_, *f*_1_*x*_2_ and *s*_1_, with *x*_1_ sites distributed along the entire alignment. Lastly, migration in both directions will produce both types of clusters.

As proposed above, an alignment can be summarized as a sequence that represents the order of the different categories of polymorphic sites (Figure 1E). This time, our objective is to independently detect (i) clusters of *x*_2_, *f*_1_*x*_2_ and *s*_1_ sites, and (ii) clusters of *x*_1_, *f*_2_*x*_1_ and *s*_2_ sites within the alignment (as candidates of introgression blocks). In order to test such patterns we will focus on the *W* statistic [37] which can be used to test for a uniform distribution of the division of a continuous interval into sub-intervals, i.e. the random position (following a uniform probability distribution) of breaks on the continuous interval. The *W* statistic was modified for the discrete case in ref [38], in order to be able to apply it to molecular sequences. In contrast with the previous *R* statistic, the modified *W* statistic is based on the length of the sub-intervals from sites of a given category (0’s) delimited by the positions of the sites of the other category (the ‘breaks’, i.e. the 1’s): $W=0.5\sum_{i=1}^{d+1}\left|\frac{l_{i}}{\left(k-2\right)}-\frac{1}{d+1}\right|$ where *k* is the total number of variable sites in the summary sequence, *d* is the number of sites assigned to category 1 and *l*_*i*_ is the length of the *i*^th^ sub-interval (there are *d* + 1 sub-intervals, including those of length zero). Thus, the category 0 is used to define the segment and the *W* statistic is sensitive to the randomness of the distribution of 1’s. This is an important characteristic of this statistic as it will allow testing for the randomness of one category of site regardless of the other category being distributed randomly or not (so the other category can be tested conversely on exchanging 1’s and 0’s of the sequence). The *W* statistic will take large values when the ‘breaks’ are clustered and low values when the ‘breaks’ are evenly spaced. This statistic can be applied to our problem by coding the alignment in two ways: (i) *x*_1_, *f*_2_*x*_1_ and *s*_2_ sites as ‘breaks’ (1’s), while *x*_2_, *f*_1_*x*_2_ and *s*_1_ define the segment (0’s) (Figure 1E; statistic *W*_*x*1*s*2_) and (ii) *x*_2_, *f*_1_*x*_2_ and *s*_1_ sites as ‘breaks’ (1’s), while *x*_1_, *f*_2_*x*_1_ and *s*_2_ define the segment (0’s) (Figure 1E; statistic *W*_*x*2*s*1_). Preliminary work on a variant of the *W* statistic including fixed differences yielded similar results (data not shown). It will thus not be presented in this work. We predict that the presence of migration from P2 to P1 (bidirectional migration or unidirectional migration from P2 to P1) will produce larger values in the statistic *W*_*x*1*s*2_ than scenarios without migration from P2 to P1 (isolation model and unidirectional migration from P1 to P2). Note that direction of migration is always indicated forward in time throughout the text, including for coalescent simulations.

In the simulations, models with populations connected only by migration (bidirectional migration and unidirectional migration from P2 to P1) were detected at all differentiation levels, though the power was higher with higher levels of genetic differentiation (Figure 2B). Unlike the *R*_*F*_ statistic, a minimum recombination rate is not necessary and the simulations without recombination show some (low) power to detect directional migration (Figure 3B). This might be due to a pattern produced by an asymmetry in the populations since higher polymorphism in the sink population than in the source population is expected. Still, recombination plays an important role for this statistic since its power increases with recombination. It is interesting to note that the presence of *S* sites for the highest differentiation levels considered was very low or null. Therefore, the values of the *W*_*x1s2*_ statistic depend only on the distribution of *X*_1_ and *X*_2_ sites and the orientation of the mutations is not required.

**Figure 4:**
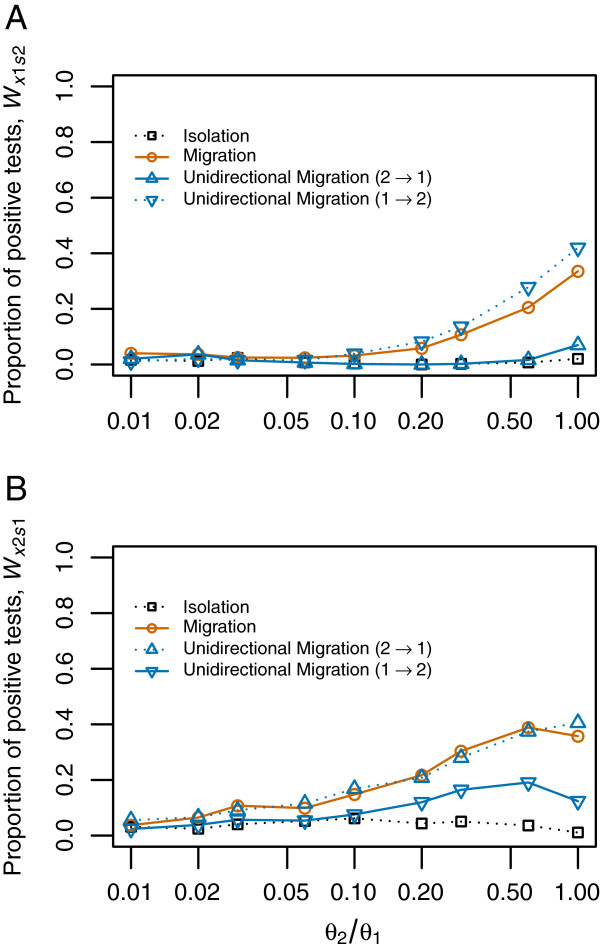
**Effect of unequal population size on the detection of unidirectional gene flow (isolation with unequal population size as null model).** 1,000 coalescent simulations were performed for each value of the parameter *θ*_2_ and each model: isolation (black), migration (red) and unidirectional migration (blue). Continuous lines indicate the proportion of significant tests under the models with migration from P2 to P1 (*W*_*x*1*s*2_ statistic) or from P1 to P2 (*W*_*x*2*s*1_ statistic), i.e. they indicate the power of the test. Dotted lines indicate the proportion of significant tests under the models without migration from P2 to P1 (*W*_*x*1*s*2_ statistic) or from P1 to P2 (*W*_*x*2*s*1_ statistic), i.e. they indicate the false positive rate. Proportion of significant tests is estimated as a function of population size ratios between P2 and P1*.***A.***W*_*x1s2*_ statistic, **B.***W*_*x2s1*_ Statistic.

The false positive rate was near, or below, the expected nominal level (5%) as neither the isolation nor the unidirectional migration from P1 to P2 produced any significant clustering of *X*_1_ and *s*_2_ sites (Figure 2C). However, as noted above, the *W*_*x1s2*_ statistic seems to be affected by asymmetries of the model other than the gene flow. Further simulations with unequal population sizes and gene flow from the small to the large populations show an extremely high false positive rate when the isolation model with equal population sizes is assumed as null model. On the other hand, when gene flow is from the large population to the small one the test is conservative but has virtually no power (Additional file 1: Figure S6). The difference in population sizes increases the differences in polymorphism between both populations, resulting in unbalanced number of *X*_*1*_ and *X*_*2*_ sites that probably affects the *W* statistics. These problems can be partially solved by using the isolation model with unequal population sizes as null model (see Methods for details). By doing so, false positives from simulations without any gene flow remain at the nominal level (Figure 4). However, signal for direction of migration is lost since, as simulations with gene flow from P2 to P1 show no significant *W*_*x1s2*_ values while simulation with gene flow from P1 to P2 yield significant *W*_*x1s2*_ values (i.e. the opposite of the desired behaviour). These results indicate that it is difficult to disentangle the effect of unidirectional gene flow from those of unequal population sizes with our test-based approach. However, more positively, *W* statistics can still offer some evidence for migration if not for their direction.

### Detection of migration within the *Drosophila simulans* species complex

The statistics *R*_*F*_, *W*_*x1s2*_ and *W*_*x2s1*_ were calculated for a *D. simulans* complex dataset (Table 1), with the aim of testing for gene flow, and assessing direction, between these closely related species. In all pairwise comparisons, the two populations of *D. mauritiana* were considered separately because of their high differentiation [27], and intraspecific comparisons were done for this species. Table 2 gives estimates of the mutation and recombination parameters for each locus and each species. For all loci, *D. simulans* showed the highest estimates for both parameters, the estimates were much lower for *D. sechellia* and intermediate values were found for the two populations of *D. mauritiana*. Compared to the parameters used in the simulations we can see that the estimated rate of recombination is high enough ($\hat{\rho }$ >10 for most locus-population combinations) to provide some power for the detection of gene flow with the statistics proposed in this work (Figure 3). For *D. simulans* and *D. mauritiana*, scaled mutation rate estimates are also of the same order of magnitude as for the simulations, but not for *D. sechellia* which shows a much smaller effective size. The level of genetic differentiation, *D*, is presented for each locus and each pairwise comparison of species in Table 3 and populations in Table 4.

**Table 1:** **Description of genetics markers and number of sequences used in the *Drosophila *** **analyses**

**Locus**	**Chromosomal location**	**Maximum length**	**Number of sequences**			
			***D. simulans***	***D. sechellia***	***D. mauritiana *** **(Mauritius Is.)**	***D. mauritiana *** **(Rodrigues Is.)**
*amyrel*	2R	1598	38	88	48	38
*joc*	3R	702	46	63	48	38
*notch*	X	1092	29	30	43	24
*obp 57d/e*	2R	2039	38	54	24	16
*odysseus*	X	843	30	29	37	25
*otu*	X	1394	31	83	37	25
*period*	X	616	38	94	38	24
*pgd*	X	2323	31	79	37	25
*sqh*	X	1816	30	82	37	24
*vermilion*	X	1477	32	85	37	24
*white*	X	1248	31	82	43	25

**Table 2:** Mutation and recombination parameter estimates

	***D. simulans***		***D. sechellia***		***D. mauritiana***			
					**Mauritius Is.**		**Rodrigues Is.**	
	${\hat{\theta }}_{\pi }$	$\hat{\rho }$	${\hat{\theta }}_{\pi }$	$\hat{\rho }$	${\hat{\theta }}_{\pi }$	$\hat{\rho }$	${\hat{\theta }}_{\pi }$	$\hat{\rho }$
*amyrel*	39.85	37.62	1.78	24.63	18.65	>100	19.73	33.08
*joc*	12.97	>100	0.09	n.a.	10.56	>100	6.02	4.15
*notch*	27.01	78.35	0.64	5.85	7.12	>100	5.62	2.78
*obp 57d/e*	68.39	47.41	0.90	1.33	20.02	>100	17.45	16.64
*odysseus*	5.72	8.36	1.54	1.55	3.03	9.33	0.73	1.10
*otu*	15.47	37.45	0.93	6.79	10.11	>100	6.01	8.4
*period*	10.12	74.82	0.73	>100	4.63	>100	4.15	1.27
*pgd*	24.07	92.35	1.79	5.53	18.51	>100	9.27	1.97
*sqh*	20.99	53.87	1.67	10.04	17.57	71.64	14.46	9.21
*vermilion*	21.63	>100	0.32	14.93	16.62	>100	12.33	0.53
*white*	15.07	85.56	1.42	0.03	7.13	>100	6.86	0.42

**Table 3:** Pairwise genetic differentiation between species and clustering-detection statistics

	***D***^***a***^	***R***_***F***_	***p*-value**	***W***_***x*** **2 ** ***s *** **1**_	***p*-value**	***W***_***x*** **1 ** ***s *** **2**_	***p*-value**
A *D. simulans* (P1) &*D. mauritiana* Mauritius Is. (P2)							
*amyrel*	11.11	1	n.a.	0.50	0.31	0.58	0.57
*joc*	2.95	1	n.a.	0.59	0.06	0.54	0.38
*notch*	7.60	1	n.a.	0.51	0.28	0.57	0.59
*obp 57d/e*	45.71	25	0.35	0.50	0.16	0.80	0.44
*odysseus*	13.76	12	0.02	0.50	0.55	0.54	0.60
*otu*	16.46	3	0.03	0.52	0.50	0.56	0.22
*period*	4.76	3	0.66	0.53	0.12	0.68	0.33
*pgd*	4.92	1	n.a.	0.55	0.18	0.51	0.57
*sqh*	10.33	3	0.40	0.53	0.13	0.59	0.66
*vermilion*	5.99	1	n.a.	0.48	0.54	0.55	0.74
*white*	5.69	1	n.a.	0.55	0.06	0.65	0.08
B *D. simulans* (P1) &*D. mauritiana* Rodrigues Is. (P2)							
*amyrel*	16.61	7	0.78	0.48	0.41	0.75	0.55
*joc*	3.96	1	n.a.	0.53	0.04	0.77	0.62
*notch*	8.17	1	n.a.	0.37	0.73	0.77	0.58
*obp 57d/e*	45.71	25	0.38	0.47	0.36	0.87	0.51
*odysseus*	13.80	12	0.02	0.63	0.01	0.90	0.51
*otu*	17.60	2	0.0004	0.4	0.53	0.83	0.28
*period*	4.46	1	n.a.	0.37	0.69	0.80	0.54
*pgd*	6.83	1	n.a.	0.44	0.33	0.78	0.41
*sqh*	11.52	5	0.46	0.43	0.56	0.86	0.27
*vermilion*	10.80	5	0.71	0.51	0.07	0.83	0.52
*white*	5.47	1	n.a.	0.33	0.90	0.81	0.29
C *D. simulans* (P1) &*D. sechellia* (P2)							
*amyrel*	32.17	31	0.71	0.46	0.19	0.95	0.70
*joc*	11.42	7	0.08	0.36	0.30	0.98	0.40
*notch*	9.82	3	0.02	0.41	0.20	0.97	0.35
*obp 57d/e*	24.88	19	0.75	0.40	0.32	0.97	0.59
*odysseus*	14.11	1	n.a.	0.40	0.29	0.87	0.60
*otu*	17.15	23	0.75	0.33	0.48	0.95	0.48
*period*	6.19	5	0.58	0.30	0.72	0.89	0.59
*pgd*	18.08	1	n.a.	0.45	0.13	0.95	0.54
*sqh*	9.53	1	n.a.	0.36	0.35	0.97	0.57
*vermilion*	16.60	18	0.63	0.36	0.33	0.97	0.75
*white*	10.82	9	0.63	0.45	0.15	0.94	0.52

^a^ pairwise genetic differentiation [35].

**Table 4:** **Pairwise genetic differentiation between populations of *D. mauritiana***^**a **^**and clustering-detection statistics**

	***D***^***b***^	***W***_***x2s1***_	***p*-value**	***W***_***x1s2***_	***p*-value**
*amyrel*	3.59	0.72	0.39	0.49	0.29
*joc*	0.71	0.81	0.42	0.48	0.16
*notch*	1.11	0.79	0.23	0.44	0.52
*obp 57d/e*	0.78	0.79	0.01	0.58	0.01
*odysseus*	0.15	0.94	0.35	0.59	0.04
*otu*	2.09	0.83	0.62	0.36	0.75
*period*	0.47	0.65	0.58	0.41	0.63
*pgd*	1.99	0.80	0.33	0.42	0.49
*sqh*	0.92	0.81	0.27	0.38	0.89
*vermilion*	3.78	0.88	0.28	0.41	0.54
*white*	1.00	0.73	0.35	0.47	0.30

^a^*D. mauritiana* Rodrigues Is. (P1) &*D. mauritiana* Mauritius Is. (P2).

^b^ pairwise genetic differentiation [35].

We found some evidence, though not definitive, for migration between *D. simulans* and its two sister species. The *R*_*F*_ statistic was significant for two loci (of five testable loci, Table 3; note that absence of one site category implies that there is a single run) for the *D. simulans*/*D. mauritiana* from Mauritius Island (Mau) pair; for two loci (of six) for the *D. simulans*/*D. mauritiana* from Rodrigues Island (Rod) pair; and for one locus (of eight) for the *D. simulans*/*D. sechellia* pair. Additional support came from the *W* statistic (note that in this case it will be considered uninformative for the direction since unequal population sizes were assumed in the null model): the two loci had significant *W*_x2s1_ values for the *D. simulans*/Rod pair. Our results, based on the spatial arrangement of polymorphisms, are consistent with a genome-wide comparison of one individual from *D. simulans*, *D. sechellia* and *D. mauritiana*, which shows that both autosomal and X-linked regions present a signal consistent with recent introgressions between *D. simulans* and the two endemic species [16]. Other studies based on smaller genomic regions have revealed that introgression was likely to have occured between *D. simulans* and Mau both at the mitochondrial and at the nuclear level [16,27,39], but our analyses are the first to suggest that introgression may also have occurred between *D. simulans* and Rod. *D. simulans* is absent from both Mauritius and Rodrigues although very common in the neighbouring island of La Réunion ([40]; D. Legrand, D. Lachaise and M-L Cariou unpublished observations). Thus, recent introgression may have two non-exclusive origins: (i) *D. simulans* was present on Mauritius and Rodrigues and recently disappeared and (ii) in the recent past, *D. simulans* arrived to both islands through human activities, fruit dispersal or climatic factors. The latter scenario opens the possibility that the *D. mauritiana* gene pool may have absorbed occasional or regular waves of *D. simulans* dispersers, without the stable presence of a separate *D. simulans* population on Mauritius. Notice that the shared history of the two *mauritiana* taxa [27] can also explain the presence of *simulans*-like alleles in Rod as a consequence of introgression from *D. simulans* into Mau that occurred before the split of the two *D. mauritiana* populations about 100,000 years ago. The situation between *D. simulans* and *D. sechellia* is different because the two species coexist on the Seychelles archipelago, and a few morphological hybrids of *D. simulans*-*D. sechellia* have been observed (D. Lachaise, personal communication).

The intraspecific comparison of the two populations of *D. mauritiana* (Table 4) cannot benefit from the information provided by *R*_*F*_ statistic since the absence of fixed differences prevents its calculation. However, one of eleven *W*_*x2s1*_ statistics was significant, and two of eleven *W*_*x1s2*_ were significant, which provides some evidence that points towards the presence of gene flow. Likelihood-based inferences under the Isolation with Migration model have previously suggested the presence of limited gene flow from Mau to Rod [27].

## Conclusions

This works confirms the prediction that the spatial arrangement of polymorphisms along a recombining stretch of a genome is affected by the presence and direction of gene flow. Two summary statistics are described that are sensitive to the spatial clustering of polymorphic sites generated by gene flow, and which can be used in a test to reject the Isolation model as null hypothesis. A third statistic is described that has a distinct behaviour in the presence of migration, but that was not adequate for the test-based approach described in this work. The interest of these statistics is that they are applicable to datasets with characteristics that prevent their analysis with current available programs such as IM [41], because of the presence of intragenic recombination, or MIMAR [8], because of the absence of fixed differences and shared polymorphisms at the same time. However, these statistics should not be seen as a substitution of those methods but rather as a complement. They allow the extraction of information from data that derives from linkage disequilibrium among alleles from recombining loci. This is information that is usually lost when IMa is applied to short sequences (length selected to assure lack of recombination within them) or in likelihood-free methods (such as approximate Bayesian computation, ABC) that do not use heavy computing LD summary statistics. These statistics can be applied to an entire dataset as a previous step before applying IMa to a reduced dataset. They may also be useful as summary statistics in the ABC framework; like the variance of pairwise differences that have low power when applied as a test [3] but have proved useful for ABC [42].

Another advantage of the statistics described in this work is that genotypes do not need to be phased nor individual genotypes identified, because the statistics depend only on the spatial location of sites not on the allelic identity of individuals. Unphased sequence data or sequence data from pooled samples of several individuals (pooling within each population) should be as informative as phased resequencing data (regarding the presently discussed statistics). Another advantage is that knowledge of allele frequencies or the ancestral state is not necessary (although *W*_*x1s2*_ was described using this information to classify *s*_1_ and *s*_2_ sites, the statistic may be calculated exclusively from *X*_1_ and *X*_2_ sites, as discussed above). This could be particularly helpful for the study of next generation sequencing data derived from pooled individuals e.g. [43], where the number of individuals sequenced at each polymorphic site and each allelic state is unknown and methods based on the PAC-likelihood or the length of shared haplotypes cannot be applied. As long as the coverage of the sequencing is enough to determine whether the site is polymorphic in each population, *R* and *W* statistics may be calculated.

The application of these statistics to the species of the *D. simulans* complex was illustrative. Although the high recombination rates (Table 2) make it an especially favourable case study for the application of these summary statistics, the results were not as compelling as they could have been. The results suggest genetic exchange between species and also between populations, that is, in the absence of any fixed differences. This conclusion is also supported by several independent studies on this system. The confirmation of the presence of gene flow is an important key for the understanding of the evolution of these species. However, we could not ascertain the presence and direction of migration due to limited power of our statistics in case of unequal population sizes. Resolving issue will be a major step toward a better comprehension of the evolutionary history of the *simulans* complex.

## Methods

### Models and simulations

Significance of each observed statistic was estimated from the distribution of the statistic under the null model (isolation model). The distribution of the test statistic was obtained by coalescent simulation [44] with recombination, with parameter values estimated from the data (see below). The isolation model (see Additional file 1: Figure S1) consists of two populations (P1 and P2, with same effective size), with no exchange of migrants, that have diverged for a period of time from a common ancestral population (with effective size equal to that of the present day populations). This model is characterized by three parameters: (i) the population-scaled mutation rate *θ* = 4*Nμ* (where *N* is the effective population size and *μ* is the mutation rate per locus per generation), (ii) the population-scaled time of divergence *T* = *t*/(2 *N*) (where *t* is the divergence time expressed in generations), and (iii) the population-scaled recombination rate *ρ* = 4*Nc* (where *c* is the recombination rate per locus per generation). Parameters *θ* and *T* for this model were estimated from the data by using the average number of pairwise differences within (*d*_*x*_ and *d*_*y*_) and between (*d*_*xy*_) populations [3]. Recombination parameter for *Drosophila* data was estimated with LDhat ([45]; see below) while the true value was taken for simulated data (assuming a good estimate could be obtained, since properly applying LDhat for each simulation was computationally infeasible). In some instances, a more complex null model was also used with unequal population sizes. This model has one scaled mutation rate parameter for each population (*θ*_1_, *θ*_2_ and *θ*_*A*_, respectively for populations P1, P2 and ancestral; note that in all our simulations *θ*_*A*_ = *θ*_1_). When unequal population sizes were used as the null model, parameter values were estimated from the number of *F*, *S*, *X*_1_ and *X*_2_ sites [30] with J. Hey’s Sites software (http://bio.cst.temple.edu/~hey/software/software.htm). Sites software numerically solves the equations of the expected number of *F*, *S*, *X*_1_ and *X*_2_ sites in function of *θ*_1_, *θ*_2_, *θ*_*A*_ and T for the observed number of *F*, *S*, *X*_1_ and *X*_2_ sites.

Power and the false positive rate of the proposed statistics were evaluated on datasets generated with coalescent simulations. Six demographic models were used for this evaluation: the isolation (I), migration (M), unidirectional migration (UM), isolation-with-migration (IM), isolation-with-unidirectional-migration (IUM) and panmictic population (P) models (see Additional file 1: Figure S1). The isolation model is as described above. The migration model (also known as two-island model) consists of two populations (with the same effective size) that have never been in contact except for a constant rate of symmetric migration. The parameters of this model are: (i) the population-scaled mutation rate *θ*, (ii) the population-scaled migration rate *M* = 4 *Nm* (where *m* is the migration rate per generation), and (iii) the population-scaled recombination rate *ρ*. The unidirectional migration model is the same as the migration model, but with migration only occurring from one population to the other. The isolation-with-migration and -with-unidirectional-migration are intermediate models with both parameters of divergence (*T*) and migration (*M*), with migration occurring between the two derived populations after the split of the ancestral population. The panmictic population corresponds to a single population of size *θ* with a sample of genes divided randomly in two. Unequal population size was also considered for I, M and UM models (see Additional file 1: Figure S2), requiring an additional parameter for each population (scaled mutation rates *θ*_1_ and *θ*_2_).

For each model, at least 14,000 pseudo-samples were generated by coalescent simulation with *ms*[44]. Each pseudo-sample consisted of a sample of 40 gene copies for each population (in the panmictic model 80, divided into two groups). Mutations were simulated according to an infinitely-many sites mutation model. Parameter values were fixed for scaled mutation and recombination rates to *θ* = 10 and *ρ* = 10 for the first set of simulations. Divergence time and migration rate parameter values were taken randomly from the intervals (0.004–40) for *M* and (0.0125–125) for *T* (see Additional file 1: Table S1). The range of values used for *M* and *T* allowed for simulations with a range of genetic differentiation representing from low-divergence populations of the same species (*D* ≈ 0) to highly differentiated species (*D* ≈ 300), and also similar levels of differentiation between divergence and migration models. A second set of simulations was produced to study the effect of recombination rate. For these simulations only I, M and UM models were used. Migration rate was fixed to *M* = 0.04 and divergence time to *T* = 12.5 (expected *D* = 135, for I and M models without recombination [3]). Recombination rate (*ρ*) took the following values: 0, 0.1, 0.2, 0.3, 0.6, 1, 2, 3, 6, 10, 20, 30, 60, 100, 200 and 300 (with 1000 simulations for each value) (see Additional file 1: Table S2). A third set of simulations was produced to study the effect of unequal population size. For these simulations only I, M and UM models were used. Migration rate was fixed to *M* = 0.04, divergence time to *T* = 12.5, recombination rate to *ρ* = 100 and scaled mutation rate for population P1 to *θ*_1_ = 10. Scaled mutation rate for population P2 to (*θ*_2_) took the following values: 0.1, 0.2, 0.3, 0.6, 1, 2, 3, 6 and 10 (with 1000 simulations for each value) (see Additional file 1: Table S3).

The differentiation statistic *D* = *d*_*xy*_-(*d*_*x*_ + *d*_*y*_)/2 (often referred to as Nei’s net distance or *D*_*a*_[35]) was calculated for each simulation to compare simulations from different models (and experimental data) with similar levels of differentiation. Polymorphic sites were classified into categories (i.e. *F*, *S*, *X*_1_ and *X*_2_; *f*_1_, *f*_2_, *s*_1_, *s*_2_, *x*_1_, *x*_2_, *f*_2_*x*_1_ and *f*_1_*x*_2_) and the statistics *R*_*F*_, *R*_*S*_, *W*_*x1s2*_ and *W*_*x2s1*_ were calculated from the alignment for each replicate. Power and false positive rates were calculated as the proportion of significant tests (with nominal level α = 0.05) under the different models. For the detection of migration with *R*_*F*_ and *R*_*S*_ statistics, M, UM, IM and IUM models were used to estimate power and I model (the null model) was used to estimate the false positive rate; while for the detection of unidirectional migration with *W*_*x*1*s*2_ statistic, M, UM (from P2 to P1), IM and IUM (from P2 to P1) models were used to estimate power and I, UM (from P1 to P2) and IUM (from P1 to P2) models were used to estimate the false positive rate. For the first set of simulations, power and false positive rate were estimated as a function of *D* (for each value *D*_*i*_ of *D* from the simulations, all simulations with *D*_*j*_ within interval [*D*_*i*_-0.2*D*_*i*_, *D*_*i*_ + 0.2*D*_*i*_] were used to estimate to estimate power at *D* = *D*_*i*_, provided there were at least 100 simulations within the interval). The proportion of significant tests for *D*_*i*_ was estimated from the simulations from the above-defined interval using a weighted (using an Epanechnikov kernel) logistic regression. For the other simulations the observed proportion of significant tests for each pre-set value of *ρ* or *θ*_1_/*θ*_2_ ratio was used. All computations were done in R [46] and the scripts are available from M. Navascués upon request.

### *Drosophila simulans* species complex

The statistics described above were applied to populations and species of the *D. simulans* complex in order to test for introgression between *D. sechellia* and *D. simulans*, between the two populations of *D. mauritiana* and *D. simulans*, and within *D. mauritiana* (Mauritius and Rodrigues populations). We specifically wanted to distinguish between the hypotheses of an absence of gene flow between all pairs of species, similar exchanges between the species or unidirectional gene flow (this later objective could not be addressed as discussed above). We extended datasets of *D. sechellia* and *D. mauritiana* used in previous studies [25,27] to construct a larger set of sequences which includes *D. simulans* and additional markers. *D. simulans* flies were representative of the species and primarily from its region of origin. They were from Madagascar, the Seychelles archipelago, Comoros, La Réunion, South Africa, Uganda, Tanzania and Annobon Island (Guinean gulf, West Africa; Additional file 1: Figure S4). *D. sechellia* flies originated from Aride, Denis, Silhouette, Coco, Cousin, Cousine, Frégate, Mahé and Praslin islands of the Seychelles archipelago. Finally, the *D. mauritiana* sample consisted of two distant populations, the Mau population from Mauritius and the Rod population from the island of Rodrigues, as in [27]. Flies were collected from the wild and directly conserved in absolute alcohol until sequencing, with the exception of some *D. simulans* lines from Madagascar and Comoros that have been kept in the laboratory for 20 to 200 generations. Experimental procedures comply with institutional, national and international ethical guidelines. Drosophila were collected with the permission of the National Parks and Conservation Service of the Ministry of Agro Industry Food Production and Security of Mauritius, the Ministry of Environment and conservation department of the Seychelles, The Ministry of Environnement of Madagascar. The global dataset consisted of 11 nuclear genes, *amyrel*, *joc*, *notch*, *obp57d/e*, *odysseus*, *otu*, *period*, *pgd*, *sqh*, *vermilion* and *white*, representing a total of 15 kb, for at least 29 gene copies per species (Table 1). Sequencing was performed following [25] and primers for each locus and each species are available upon request. Sequences were aligned by visual inspection using Bioedit v7.0.9.0; estimates of the scaled recombination rate, *ρ*, for each locus and taxon were performed by the composite likelihood method implemented in LDhat [45] (for estimates of *ρ >* 100 a value of *ρ =* 100 was used in the null hypothesis), and estimates of scaled mutation rate from nucleotide polymorphism, *θ*_*π*_, were computed with DnaSP v5 [47]. Polymorphic sites were classified in categories (i.e. *F*, *S* and *X*; *s*_1_, *s*_2_, *x*_1_, *x*_2_, *f*_2_*x*_1_ and *f*_1_*x*_2_), and the statistics *R*_*F*_, *R*_*S*_, *W*_*x2s1*_ and *W*_*x1s2*_ were calculated from the alignments. Given the clear differences in size among populations, significance of the statistics was tested using the isolation models with unequal population sizes as null model. Ancestral and derived states were deduced by the use of *D. melanogaster* as an outgroup, for which sequences were obtained from the whole genome of *D. melanogaster* available in GenBank under the following accession numbers: AE014298 (chromosome X), AE013599 (chromosome 2R), AE014297 (chromosome 3R). The *Drosophila* sequence data set supporting the results of this article is available in the Dryad Digital Repository, with identifier doi: 10.5061/dryad.67f8v (http://doi.org/10.5061/dryad.67f8v).

## Competing interests

The authors declare that they have no competing interests.

## Authors’ contributions

FD and MN conceived and developed the new statistics. MN performed the simulation work and analysed the simulated data. MLC and DL collected the *Drosophila* samples. DL and CC performed the molecular work for obtaining the DNA sequences. DL, CC and MN analysed the *Drosophila* data. MN, DL, MLC and FD wrote the article. All authors approved the final version of the article.

## Supplementary Material

Additional file 1 Supplementary Materials and Methods section and supplementary Tables and Figures. Click here for file
