# Supplementary material for: Distinguishing migration from isolation using genes with intragenic recombination: detecting introgression in the Drosophila simulans species complex
Source: BMC Evol Biol. 2014 Apr 24;14:89. doi: 10.1186/1471-2148-14-89 (PMC4022370; doi:10.1186/1471-2148-14-89)
Supplement: Additional file 1 — Supplementary Materials and Methods section and supplementary Tables and Figures. [file 1471-2148-14-89-S1.pdf]

Supplementary Document for

Distinguishing Migration from Isolation Using Genes with Intragenic Recombination: Detecting Introgression in the *Drosophila simulans* Species Complex

by Miguel Navascués, Delphine Legrand, Cécile Campagne, Marie-Louise Cariou and Frantz Depaulis

Supplementary Materials and Methods

In addition to the simulations described in the main text, three sets of simulations were performed to study the effects of sample size, scaled mutation rate ($\theta = 4N\mu$) and hierarchical substructure. The effect of sample size was studied for the isolation, migration and unidirectional migration models (see main text and figure S1) where scaled mutation rate was fixed to $\theta = 10$, recombination rate to $\rho = 10$, migration rate to $M = 0.04$ and divergence time to $T = 12.5$. Sample sizes (per population) took the following values: 2, 4, 10, 20, 40 and 100 (with 1000 simulations for each value). Results from these simulations are shown in figure S7. The effect of θ was studied for the same models (I, M and UM) with recombination rate fixed to $\rho = 10$, migration rate to $M = 0.04$, divergence time to $T = 12.5$ and 40 gene copies sampled per population; and θ values: 1, 1.5, 2, 3, 5, 7, 10, 15 and 20. Results from these simulations are shown in figure S8.

The effect of hierarchical structure was studied with alternative versions of the isolation, migration and unidirectional migration models in which one of the populations is subdivided in four demes connected by migration (see figure S3). The strength of the substructure is determined by the migration rate among those demes. The isolation model with substructure is described by nine parameters: the size of population one $\theta_1 = 10$, the size of the four demes connected by migration $\theta_2 = \theta_3 = \theta_4 = \theta_5 = 2.5$, the ancestral size $\theta_A = 10$, the divergence time $T = 12.5$, the recombination rate $\rho = 10$ and the migration rate among demes M' which took values: 0.4, 4, 40 and 400. Models with migration are defined by eight parameters: the size of population one $\theta_1 = 10$, the size of the four demes connected by migration $\theta_2 = \theta_3 = \theta_4 = \theta_5 = 2.5$, the recombination rate $\rho = 10$, the migration rate between population 1 and deme 2 $M = 0.04$ and the migration rate among demes M' which took values: 0.4, 4, 40 and 400. In the unidirectional migration model the structured population is always de source of the gene flow. Forty gene copies were sampled from the first (unstrcutured) population and 10 from each demes (2–5). Statistics were calculated pooling samples from demes 2–5 as if taken from a single population. Results from these simulations are shown in figure S9.

Model	Parameter	Values
Isolation	$\theta = 4N\mu$	10
	$\rho = 4Nr$	10
	$T = t/(2N)$	logunif(0.0125–125)
	sample size	40+40
Migration	$\theta = 4N\mu$	10
	$\rho = 4Nr$	10
	$M = 4Nm$	logunif(0.004–40)
	sample size	40+40
Unidirectional Migration	$\theta = 4N\mu$	10
	$\rho = 4Nr$	10
	$M = 4Nm$	logunif(0.004–40)
	sample size	40+40
Isolation with Migration	$\theta = 4N\mu$	10
	$\rho = 4Nr$	10
	$T = t/(2N)$	logunif(0.0125–125)
	$M = 4Nm$	logunif(0.004–40)
	sample size	40+40
Isolation with Unidirectional Migration	$\theta = 4N\mu$	10
	$\rho = 4Nr$	10
	$T = t/(2N)$	logunif(0.0125–125)
	$M = 4Nm$	logunif(0.004–40)
	sample size	40+40
Panmictic population	$\theta = 4N\mu$	10
	$\rho = 4Nr$	10
	sample size	80

Table S1: Parameter values used in the main simulation set.

Model	Parameter	Values
Isolation	$\theta = 4N\mu$	10
	$\rho = 4Nr$	(0, 0.1, 0.2, 0.3, 0.6, 1, 2, 3, 6, 10, 20, 30, 60, 100, 200, 300)
	$T = t/(2N)$	12.5
	sample size	40+40
Migration	$\theta = 4N\mu$	10
	$\rho = 4Nr$	(0, 0.1, 0.2, 0.3, 0.6, 1, 2, 3, 6, 10, 20, 30, 60, 100, 200, 300)
	$M = 4Nm$	0.04
	sample size	40+40
Unidirectional Migration	$\theta = 4N\mu$	10
	$\rho = 4Nr$	(0, 0.1, 0.2, 0.3, 0.6, 1, 2, 3, 6, 10, 20, 30, 60, 100, 200, 300)
	$M = 4Nm$	0.04
	sample size	40+40

Table S2: Parameter values used in the simulation set to study the effect of recombination.

Model	Parameter	Values
Isolation	$\theta_1 = 4N_1\mu$	10
	$\theta_2 = 4N_2\mu$	(0.1, 0.2, 0.3, 0.6, 1, 2, 3, 6, 10)
	$\theta_A = 4N_A\mu$	10
	$\rho = 4N_1 r$	100
	$T = t/(2N_1)$	12.5
	sample size	40+40
Migration	$\theta_1 = 4N_1\mu$	10
	$\theta_2 = 4N_2\mu$	(0.1, 0.2, 0.3, 0.6, 1, 2, 3, 6, 10)
	$\rho = 4N_1 r$	100
	$M = 4N_1 m$	0.04
	sample size	40+40
Unidirectional Migration	$\theta_1 = 4N_1\mu$	10
	$\theta_2 = 4N_2\mu$	(0.1, 0.2, 0.3, 0.6, 1, 2, 3, 6, 10)
	$\rho = 4N_1 r$	100
	$M = 4N_1 m$	0.04
	sample size	40+40

Table S3: Parameter values used in the simulation set to study the effect of unequal population size.

Model	Parameter	Values
Isolation	$\theta = 4N\mu$	10
	$\rho = 4Nr$	10
	$T = t/(2N)$	12.5
	sample size	(2+2, 4+4, 10+10, 20+20, 40+40, 100+100)
Migration	$\theta = 4N\mu$	10
	$\rho = 4Nr$	10
	$M = 4Nm$	0.04
	sample size	(2+2, 4+4, 10+10, 20+20, 40+40, 100+100)
Unidirectional Migration	$\theta = 4N\mu$	10
	$\rho = 4Nr$	10
	$M = 4Nm$	0.04
	sample size	(2+2, 4+4, 10+10, 20+20, 40+40, 100+100)

Table S4: Parameter values used in the simulation set to study the effect of sample size.

Model	Parameter	Values
Isolation	$\theta = 4N\mu$	(1, 1.5, 2, 3, 5, 7, 10, 15, 20)
	$\rho = 4Nr$	10
	$T = t/(2N)$	12.5
	sample size	40+40
Migration	$\theta = 4N\mu$	(1, 1.5, 2, 3, 5, 7, 10, 15, 20)
	$\rho = 4Nr$	10
	$M = 4Nm$	0.04
	sample size	40+40
Unidirectional Migration	$\theta = 4N\mu$	(1, 1.5, 2, 3, 5, 7, 10, 15, 20)
	$\rho = 4Nr$	10
	$M = 4Nm$	0.04
	sample size	40+40

Table S5: Parameter values used in the simulation set to study the effect of $\theta = 4N\mu$.

Model	Parameter	Values
Isolation	$\theta_1 = 4N_1\mu$	10
	$\theta_A = 4N_A\mu$	10
	$\theta_2 = 4N_2\mu$	2.5
	$\theta_3 = 4N_3\mu$	2.5
	$\theta_4 = 4N_4\mu$	2.5
	$\theta_5 = 4N_5\mu$	2.5
	$\rho = 4N_1r$	10
	$T = t/(2N_1)$	12.5
	$M' = 4N_1m$	(0.4, 4, 40, 400)
	sample size	40+10+10+10+10
Migration	$\theta_1 = 4N_1\mu$	10
	$\theta_2 = 4N_2\mu$	2.5
	$\theta_3 = 4N_3\mu$	2.5
	$\theta_4 = 4N_4\mu$	2.5
	$\theta_5 = 4N_5\mu$	2.5
	$\rho = 4N_1r$	10
	$T = t/(2N_1)$	12.5
	$M' = 4N_1m$	(0.4, 4, 40, 400)
	$M = 4N_1m$	0.04
	sample size	40+10+10+10+10
Unidirectional Migration	$\theta_1 = 4N_1\mu$	10
	$\theta_2 = 4N_2\mu$	2.5
	$\theta_3 = 4N_3\mu$	2.5
	$\theta_4 = 4N_4\mu$	2.5
	$\theta_5 = 4N_5\mu$	2.5
	$\rho = 4N_1r$	10
	$T = t/(2N_1)$	12.5
	$M' = 4N_1m$	(0.4, 4, 40, 400)
	$M = 4N_1m$	0.04
	sample size	40+10+10+10+10

Table S6: Parameter values used in the simulation set to study the effect of hierarchical structure.

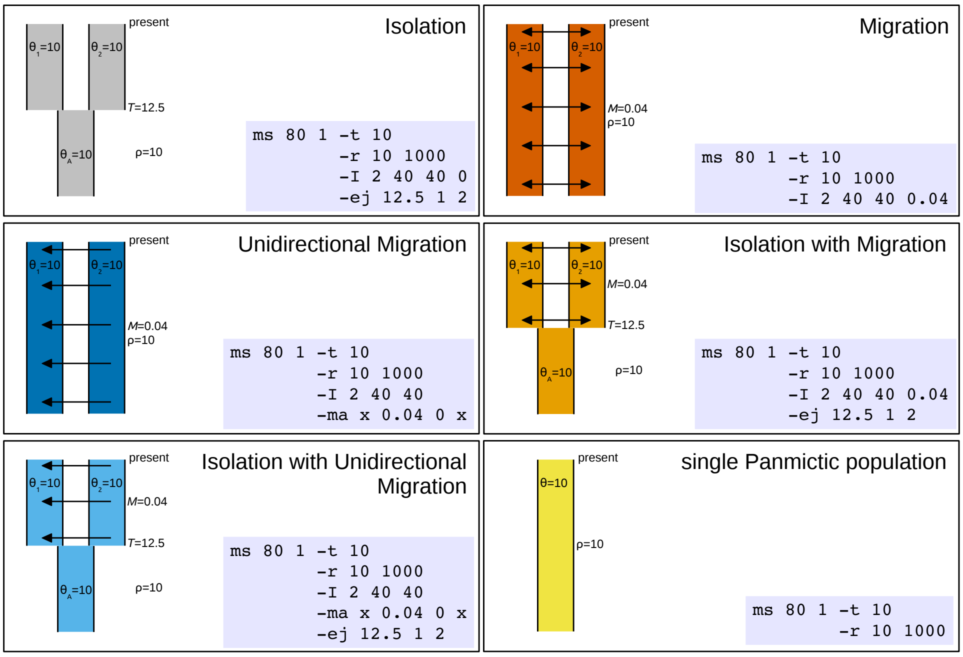

Figure S1: **Demographic models used in the simulations**. Six different demographic models were simulated to asses the performance of the tests described in the main text. The graphical representation of each model shows shaded areas representing the population size through time (with present time at the top and past at the bottom). Colour of the areas is chosen to match the colours used at the other figures. Migration is represented by horizontal arrows at arbitrary time points but it can occur at any time. Parameters of the model are represented with example values matching those used in the example *ms* command (simulations were performed also with other values). The command used in the software *ms* (with example values) is presented next to the graphical representation of each model within a shaded area. Note that the command has been split into several lines for easier reading.

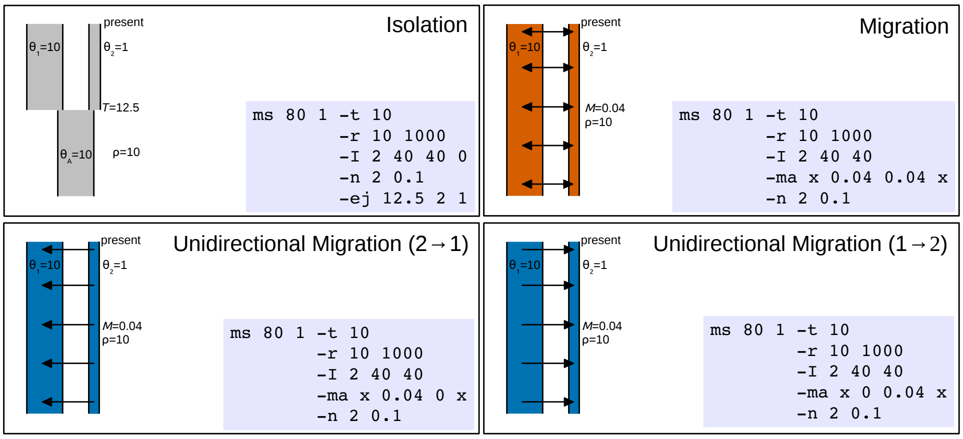

Figure S2: **Demographic models with unequal population size used in the simulations**. Four different demographic models were simulated to asses the performance of the tests on populations with unequal population sizes. The graphical representation of each model shows shaded areas representing the population size through time (with present time at the top and past at the bottom). Colour of the areas is chosen to match the colours used at the other figures. Migration is represented by horizontal arrows at arbitrary time points but it can occur at any time. Parameters of the model are represented with example values matching those used in the example *ms* command (simulations were performed also with other values). The command used in the software *ms* (with example values) is presented next to the graphical representation of each model within a shaded area. Note that the command has been split into several lines for easier reading.

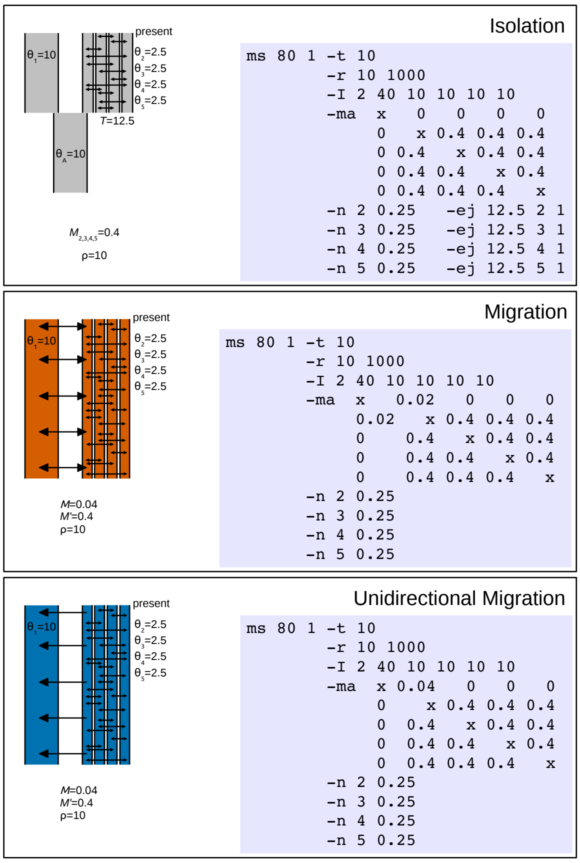

Figure S3: **Demographic models with hierarchical structure used in the simulations**. Three different demographic models were simulated to asses the performance of the tests on structured populations. The graphical representation of each model shows shaded areas representing the population size through time (with present time at the top and past at the bottom). Colour of the areas is chosen to match the colours used at the other figures. Migration is represented by horizontal arrows at arbitrary time points but it can occur at any time. Parameters of the model are represented with example values matching those used in the example *ms* command (simulations were performed also with other values). The command used in the software *ms* (with example values) is presented next to the graphical representation of each model within a shaded area. Note that the command has been split into several lines for easier reading.

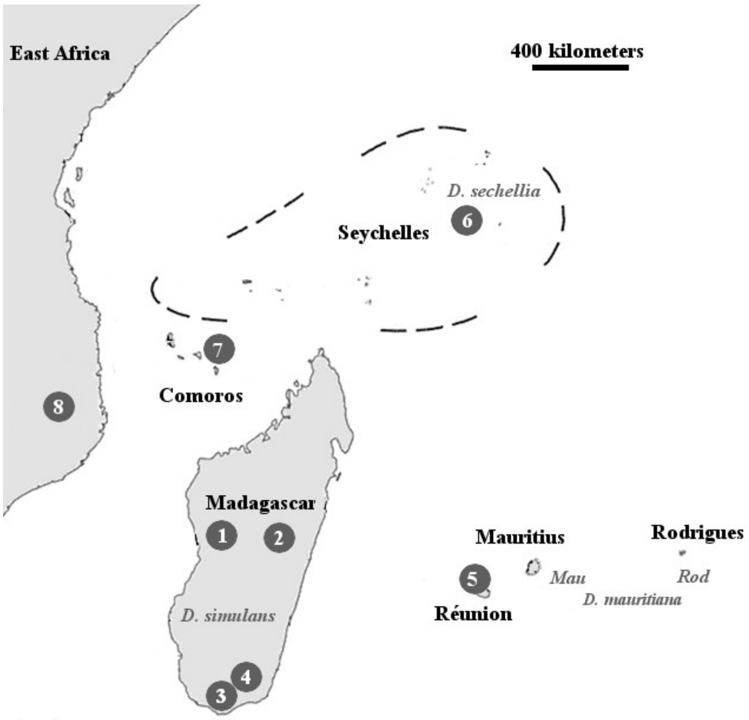

Figure S4: **The study area**. *Drosophila sechellia* flies originated from Aride, Denis, Silhouette, Coco, Cousin, Cousine, Frégate, Mahé and Praslin islands of the Seychelles archipelago. *D. mauritiana* was collected on Mauritius and Rodrigues islands. Sample of *D. simulans* were collected in 8 sites including Madagascar (1,2,3,4), La Réunion (5), the Seychelles archipelago (6), Comoros (7), Uganda (8), Tanzania (8), South Africa and Annobon Island (Guinean gulf, West Africa).

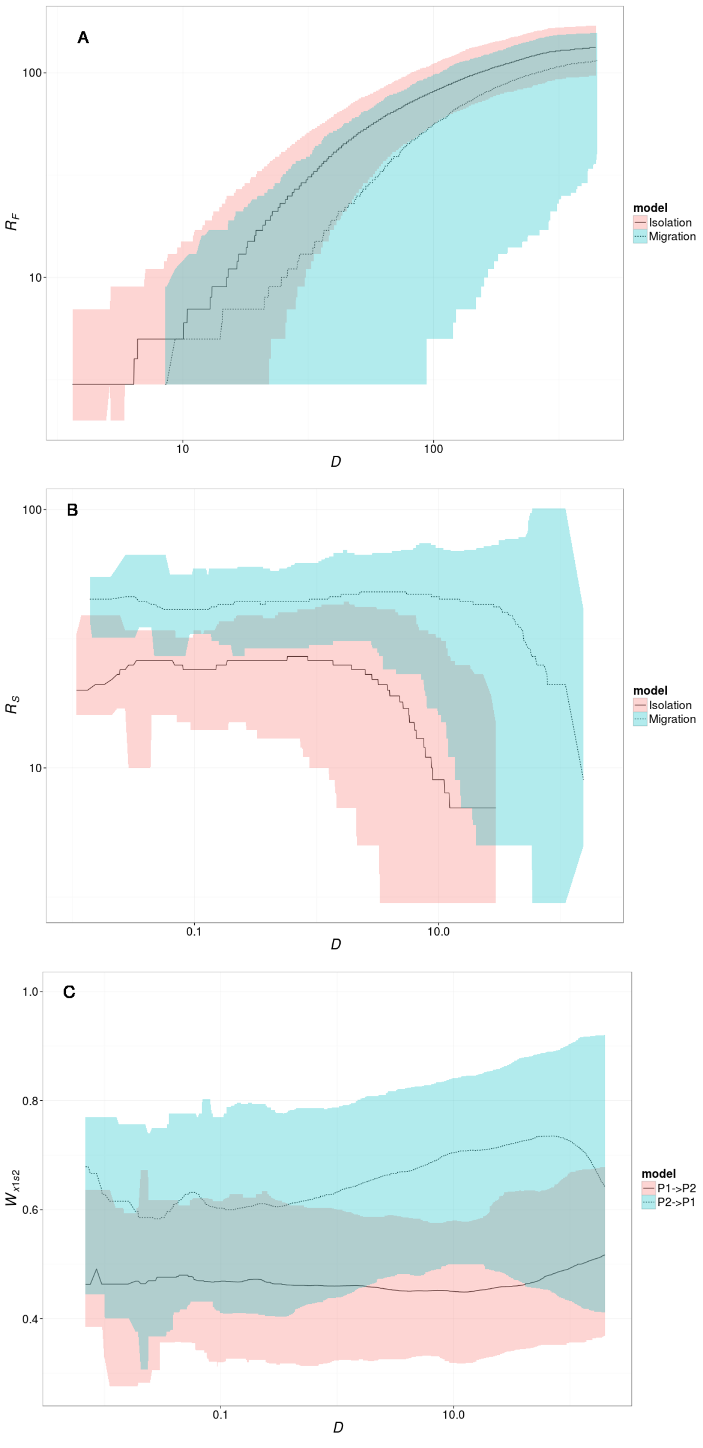

Figure S5: **Distribution of the statistic values in function of the genetic differentiation**. The 95% envelope (colored area) and median (line) of the observed statistic values in 14,000 simulations (per model) for the most contrasting pair of models for each statistic: A, comparison of Isolation (red) and Migration (blue) models for R_F; B, comparison of Isolation (red) and Migration (blue) models for R_S, and C, comparison of Unidirectional Migration from P1 to P2 (red) and Unidirectional Migration from P2 to P1 (blue) for W_{x1s2}.

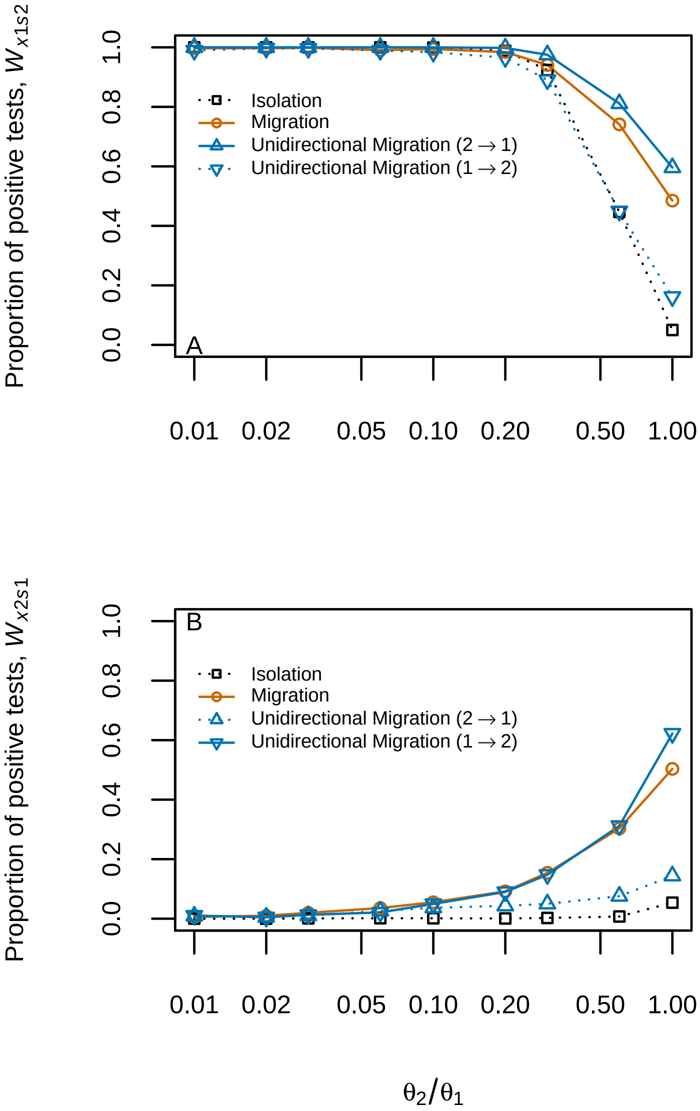

Figure S6: **Effect of unequal population size on the detection of unidirectional gene flow (isolation with equal population size as null model)**. 5,000 coalescent simulations were performed for each model: isolation (black), migration (red) and unidirectional migration (blue). Continuous lines indicate the proportion of significant tests under the models with migration from P2 to P1 (W_{x1s2} statistic) or from P2 to P1 (W_{x2s1} statistic), i.e. they indicate the power of the test. Dotted lines indicate the proportion of significant tests under the models without migration from P2 to P1 (W_{x1s2} statistic) or from P2 to P1 (W_{x2s1} statistic), i.e. they indicate the false positive rate. Proportion of significant test is estimated as a function of population size ratios between P1 and P2.

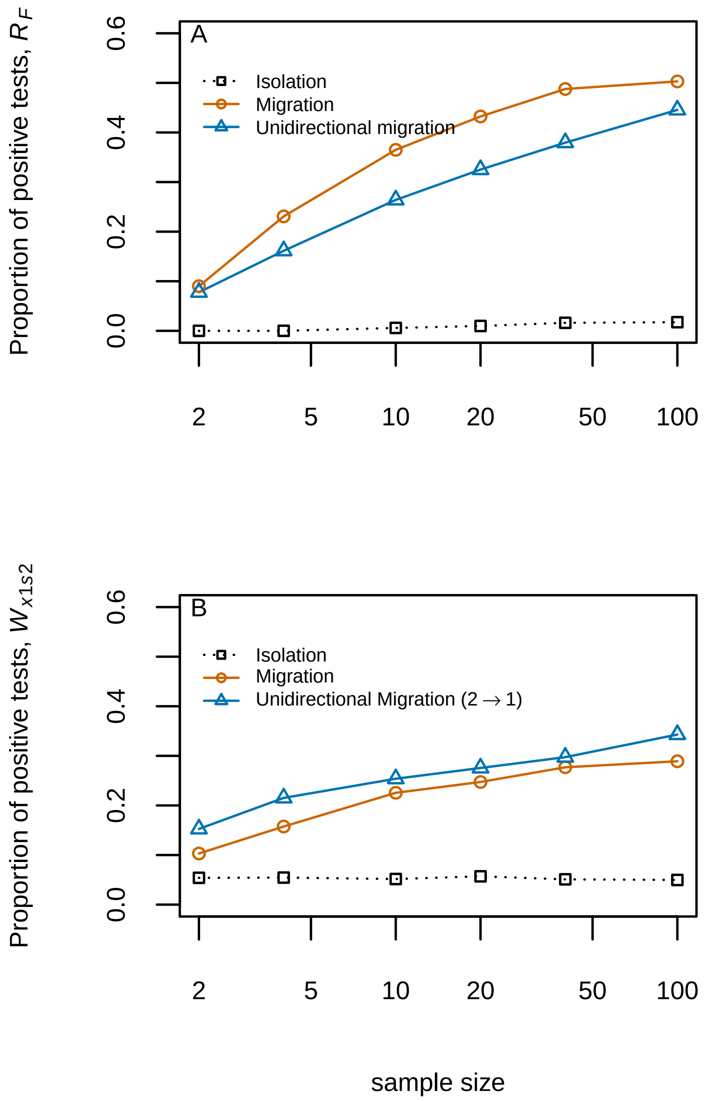

Figure S7: **Effect of sample size on the proportion of significant test**. 1,000 coalescent simulations were performed for each model: isolation (black), migration (red) and asymmetric migration (blue) and sample size value. Continuous lines indicate the proportion of significant test under the models with presence of migration (for R statistics) or presence of unidirectional migration from P2 to P1 (for W statistics), i.e. they indicate the power of the test. Dotted lines indicate the proportion of significant test under the models without migration (for R statistics) or without migration from P2 to P1 (for W statistics), i.e. they indicate the false positive rate.

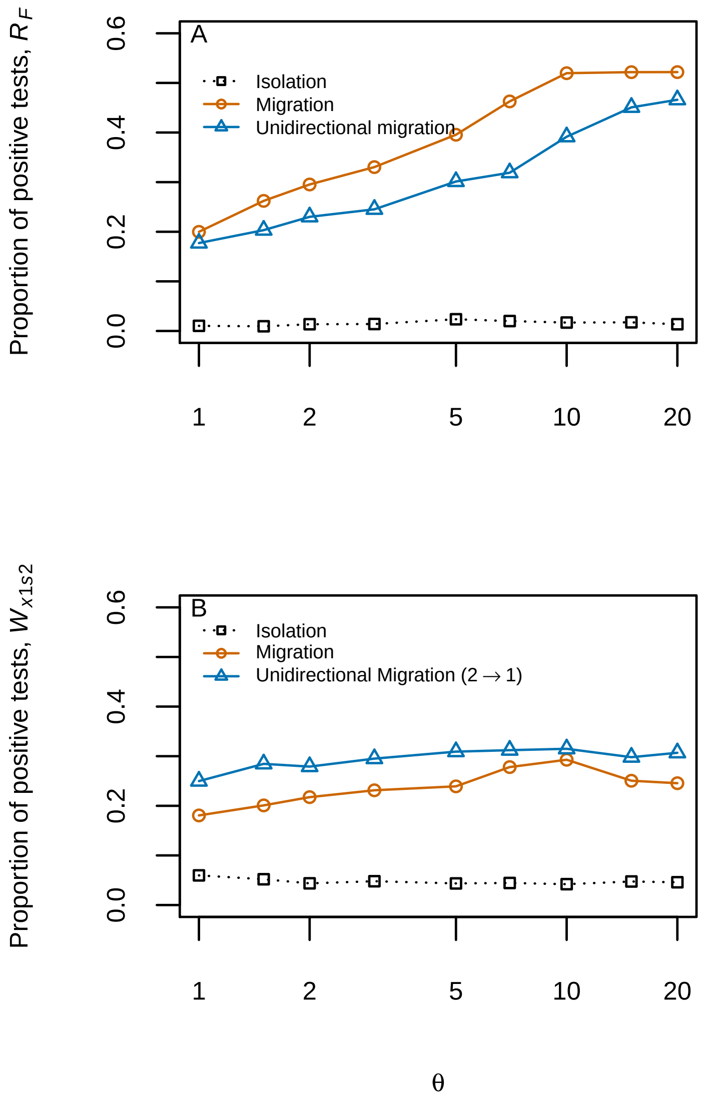

Figure S8: **Effect of $\theta = 4N\mu$ on the proportion of significant test**. 1,000 coalescent simulations were performed for each model: isolation (black), migration (red) and asymmetric migration (blue) and sample size value. Continuous lines indicate the proportion of significant test under the models with presence of migration (for R statistics) or presence of unidirectional migration from P2 to P1 (for W statistics), i.e. they indicate the power of the test. Dotted lines indicate the proportion of significant test under the models without migration (for R statistics) or without migration from P2 to P1 (for W statistics), i.e. they indicate the false positive rate.

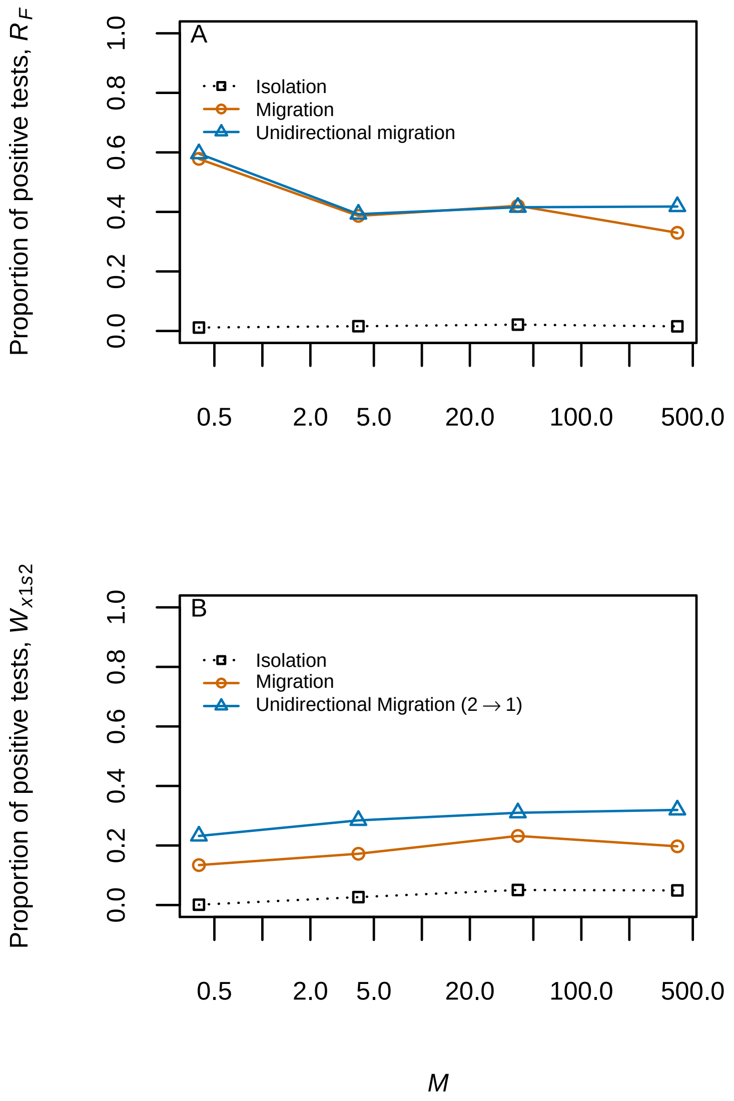

Figure S9: **Effect of hierarchical structure on the proportion of significant test**. 1,000 coalescent simulations were performed for each model: isolation (black), migration (red) and asymmetric migration (blue) and sample size value. Continuous lines indicate the proportion of significant test under the models with presence of migration (for R statistics) or presence of unidirectional migration from P2 to P1 (for W statistics), i.e. they indicate the power of the test. Dotted lines indicate the proportion of significant test under the models without migration (for R statistics) or without migration from P2 to P1 (for W statistics), i.e. they indicate the false positive rate.
